# Physicochemical Stability of Aztreonam/Avibactam in Elastomeric Devices for Outpatient Parenteral Antimicrobial Therapy

**DOI:** 10.3390/antibiotics15070708

**Published:** 2026-07-21

**Authors:** Cristina Toro Blanch, Xabier Larrea Urtaran, Raquel Aguilar Salmerón, Alba Prat Riera, Laura Gómez, Anna Costa Pou, Beatriz Fernández-Rubio, Laura Herrera-Hidalgo, Carles Quiñones Ribas

**Affiliations:** 1 Pharmacy Department, Hospital Universitari de Girona Doctor Josep Trueta, 17007 Girona, Spain; xlarrea.girona.ics@gencat.cat (X.L.U.); raguilar.girona.ics@gencat.cat (R.A.S.); albaprat.girona.ics@gencat.cat (A.P.R.); carles.quinones@gencat.cat (C.Q.R.); 2 Serveis Tècnics de Recerca, Universitat de Girona, Parc de Recerca i Innovació, 17003 Girona, Spain; laura.gomez@udg.edu (L.G.); anna.costa@udg.edu (A.C.P.); 3 Pharmacy Department, Investigation Institute i+12, Hospital Universitario 12 de Octubre, 28041 Madrid, Spain; bfernandezrubio@salud.madrid.org; 4 Unidad de Gestión Clínica de Farmacia, Instituto de Biomedicina de Sevilla (IBiS), Hospital Universitario Virgen del Rocío, 41013 Seville, Spain; laura.herrera.sspa@juntadeandalucia.es; 5 Unidad Clínica de Enfermedades Infecciosas, Microbiología y Parasitología (UCEIMP), Instituto de Biomedicina de Sevilla (IBiS), Hospital Universitario Virgen del Rocío, 41013 Seville, Spain; 6 Centro de Investigación en Red de Enfermedades Infecciosas (CIBERINFEC), Instituto de Salud Carlos III, 28029 Madrid, Spain

**Keywords:** stability, antibiotic, outpatient parenteral antimicrobial therapy, elastomers, avibactam, aztreonam

## Abstract

Background/Objectives: Outpatient Parenteral Antimicrobial Therapy (OPAT) programs provide a safe and cost-effective strategy for administering intravenous antimicrobials in the home setting. Aztreonam/avibactam (ATM/AVI) is a reserve antibiotic used to treat infections caused by multidrug-resistant Gram-negative bacteria. The aim of this study was to evaluate the physicochemical stability of ATM/AVI in elastomeric pumps for OPAT use. Methods: ATM/AVI was diluted in a 0.9% sodium chloride solution to a concentration of 25/8.33 mg/mL and stored in elastomeric pumps (Infusor LV 10 mL/h, 240 mL; Baxter Healthcare S.A, Zurich, Switzerland). The devices were maintained at 4 °C for 14 days and at 25, 32, and 37 °C for 48 h. Three independent elastomeric devices were prepared for each temperature condition and sampled in duplicate at each time point. Solutions were considered stable if the color, clarity, and pH remained unchanged and if the percentage of intact drug remained ≥90%, as determined via UHPLC-MS/MS. Results: ATM/AVI in polyisoprene elastomeric devices remained physicochemically stable for up to 14 days under refrigerated conditions and for 48 h at 25 °C, 32 °C, and 37 °C. Conclusions: ATM/AVI solutions demonstrated prolonged stability under the evaluated conditions, supporting their potential use in OPAT programs via 24 h continuous infusion.

## 1. Introduction

Outpatient Parenteral Antimicrobial Therapy (OPAT) programs provide an effective and safe strategy for administering intravenous antimicrobials outside the hospital setting, reducing hospital admissions, healthcare costs, and nosocomial complications while also improving patients’ quality of life [1,2].

Not all antimicrobial agents are suitable for OPAT, as their use depends on both pharmacokinetic/pharmacodynamic (PK/PD) characteristics and physicochemical stability. Time-dependent antimicrobials, particularly β-lactams, often require prolonged or continuous infusion to maintain adequate drug concentrations above the minimum inhibitory concentration (MIC) and optimize therapeutic target attainment. Consequently, continuous infusion regimens are especially relevant in the outpatient setting. In addition, OPAT feasibility is strongly influenced by the stability of antimicrobial solutions in elastomeric pumps, which may be affected by storage conditions, infusion duration, temperature, light exposure, and device materials [3,4].

Aztreonam/avibactam (ATM/AVI) is a fixed-dose combination of a monobactam β-lactam antibiotic and a non-β-lactam β-lactamase inhibitor developed to treat infections caused by multidrug-resistant (MDR) Gram-negative organisms. ATM/AVI exhibits potent activity against a broad range of beta-lactamase-producing Enterobacterales, including pathogens producing Class A, Class C, and certain Class D enzymes. While aztreonam is inherently resistant to hydrolysis by Class B metallo-β-lactamases, it is susceptible to degradation by other serine β-lactamases often co-produced by these organisms; the addition of avibactam neutralizes these enzymes, thereby restoring the antibacterial activity of aztreonam. For this reason, aztreonam–avibactam plays an important role in the treatment of difficult-to-treat MDR Gram-negative infections [5,6].

The standard dosage of ATM/AVI used was a loading dose of 2 g/0.67 g followed by a maintenance dose of 1.5 g/0.5 g administered intravenously over 3 h every 6 h, with dose adjustments based on renal function [7]. In the OPAT setting, continuous infusion of ATM/AVI through elastomeric devices could be an interesting alternative because its time-dependent bactericidal activity may optimize PK/PD target attainment.

Despite these favorable characteristics, due to its recent introduction into the therapeutic arsenal (approved in the EU in April 2024 and by the FDA in February 2025), physicochemical stability data that guarantee its stability in elastomeric devices under real-world storage conditions are still lacking.

Previous studies evaluating the stability of ceftazidime/avibactam in elastomeric devices have shown that avibactam is more stable than ceftazidime under the tested conditions, suggesting that avibactam degradation is unlikely to represent the limiting factor in the overall stability of the combination [8,9,10]. ATM has also demonstrated adequate stability profiles in elastomeric devices [9]. Based on these findings, we hypothesized that the combination of ATM/AVI could maintain physicochemical stability.

In this study, we aimed to evaluate the physicochemical stability of ATM/AVI in polyisoprene elastomeric devices at different temperatures for the treatment of infections caused by MDR Gram-negative organisms in OPAT programs.

## 2. Results

### 2.1. Chemical Stability

Under refrigerated conditions (4 °C), AVI and ATM maintained concentrations above 90% of their initial concentration throughout the evaluation period (14 days).

At 25 °C, both antibiotics remained stable throughout the 48 h evaluation period. ATM showed concentrations of 101.4% at 24 h and 100.3% at 48 h, whereas AVI presented values of 104.7% and 105.2%, respectively. Neither compound showed relevant degradation or losses greater than 10%.

At 32 °C, both antibiotics retained more than 90% of their initial concentration at 24 h and 48 h, with concentrations ranging from 98.3 to 97.1% for ATM and from 98.0 to 98.7% for AVI. No significant losses were observed during the 48 h period.

At the highest temperature evaluated, 37 °C, stability was slightly reduced. ATM showed a concentration of 95.2% at 24 h and 93.9% at 48 h, still remaining within the accepted stability limit of ≥90%. AVI maintained values of 99.7% at 24 h and 100.1% at 48 h, indicating good stability within this interval.

Overall, the results demonstrate that the ATM/AVI combination met the criterion for chemical stability (≥90% of the initial concentration) across the four temperature ranges studied during the time periods relevant for outpatient parenteral infusion. Mean values and 90% confidence intervals for each sampling point are summarized in Table 1.

**Figure 1:**
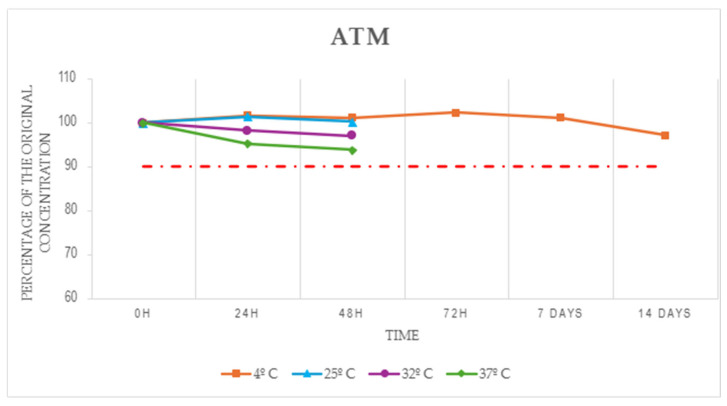
Stability of ATM (aztreonam) expressed as mean percentage of original concentration over time at 4 °C, 25 °C, 32 °C, and 37 °C.

**Figure 2:**
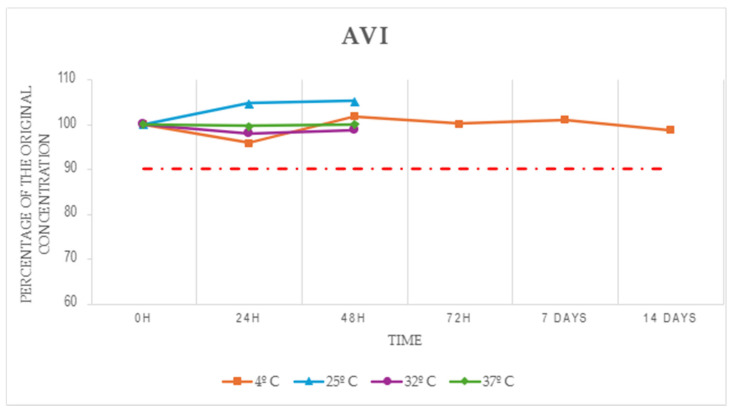
Stability of AVI (avibactam) expressed as mean percentage of original concentration over time at 4 °C, 25 °C, 32 °C, and 37 °C.

AVI and ATM solution stability data are depicted in Figure 1 and Figure 2.

### 2.2. Physical Stability

No visible precipitation was observed for any sample at any temperature, and all samples remained clear, with no visible turbidity. No color changes were observed in the ATM/AVI samples at any of the temperatures evaluated. pH values remained stable, with variations of less than one unit across all evaluated storage temperatures and time points.

Table 2 summarizes the observed values for pH, color, clarity, and precipitation at different sampling time points.

## 3. Discussion

This study shows that ATM/AVI diluted with normal saline (NS) in polyisoprene elastomeric devices could be included in OPAT programs. In contrast to other β-lactam combinations, this antimicrobial combination remains highly stable during storage under refrigerated and room-temperature conditions, as well as at body-contact temperature (32 °C) and even under elevated temperatures up to 37 °C, thereby supporting its potential use in continuous infusion administration.

The administration of ATM/AVI via continuous infusion in the OPAT setting is particularly advantageous, given its ability to maximize attainment of the PK/PD target for beta-lactams by maintaining free drug concentrations above the MIC for prolonged periods. Compared with intermittent infusion, this strategy may contribute to improved clinical outcomes. Furthermore, by minimizing periods of subtherapeutic drug exposure, continuous infusion may reduce the selection of resistant bacterial subpopulations and potentially limit the emergence of antimicrobial resistance, although clinical evidence supporting this effect remains limited [11,12,13]. Moreover, ATM and AVI have relatively short elimination half-lives (approximately 2.8 h for ATM and 2.2 h for AVI) and low protein binding (38% and 8%, respectively) and are predominantly eliminated via renal excretion. These properties result in rapid drug clearance and may increase the risk of subtherapeutic concentrations [7].

The stability of ATM/AVI for up to 14 days under refrigeration (4 °C) enables a batch preparation strategy in hospital pharmacies. This approach allows multiple devices to be prepared simultaneously and stored under refrigerated conditions. An additional advantage may be a reduction in home nursing visits, as refrigerated elastomeric devices could be stored and administered at home by trained patients or caregivers. Such a strategy optimizes resources, reduces costs associated with daily preparation, and may improve the overall quality of life of patients enrolled under the OPAT setting.

The thermal robustness demonstrated by the ATM/AVI combination, maintaining physicochemical stability for at least 48 h up to 37 °C, represents a significant advantage for home-based therapy. While standard ambient temperatures are typically 25 °C, real-world data have shown that in warmer climates or when elastomeric devices are worn close to the patient’s body, solution temperatures may frequently rise to higher levels. It is well-documented that several antimicrobials, particularly β-lactams, exhibit a significant loss of physicochemical stability at elevated temperatures [14,15].

Our findings are consistent with previously reported stability data. Fernández-Rubio et al. [9] demonstrated that ATM, tested at 12 g/L in NS, remains physicochemically stable (≥90%) throughout the 72 h at all evaluated temperatures (4 °C, 25 °C, 32 °C, and 37 °C). Stability was unaffected by the container type, with identical results observed in polypropylene infusion bags and polyisoprene elastomeric pumps. In this study, AVI was evaluated as part of the ceftazidime/avibactam combination (12/3 g/L in NS), and it remained chemically stable (≥90%) for the entire 72 h at all tested temperatures and in both container types. AVI has shown superior stability compared to its partners in other combinations, such as ceftazidime/avibactam, where the inhibitor remains stable even as the cephalosporin degrades [8,9,10].

Loeuille et al. [16] also demonstrated that ATM (at a concentration of 50 mg/mL) maintained physicochemical stability for up to 48 h at 37 °C in NS and 5% dextrose (D5W) when stored in polyisoprene elastomeric devices. AVI was also evaluated as part of the ceftazidime/avibactam combination, and the stability of the component itself was high. At 37 °C in elastomeric devices, AVI (6.25 mg/mL) remained in concentrations above the 90% threshold for 48 h in both NS and D5W.

Arlicot et al. [17] reported that ATM, tested at concentrations ranging from 1.1 to 11.3 mg/mL in NS, remained physicochemically stable for up to 72 h at 35 °C in elastomeric infusion devices, in contrast to other β-lactams that exhibited marked degradation at elevated temperatures. However, these concentrations were markedly lower than those evaluated in our study, likely because the investigation was conducted in a pediatric population.

Despite the clinical relevance of our findings, this study has several limitations that should be acknowledged. First, our analysis focused exclusively on the quantification of active pharmaceutical ingredients using ultra-high-performance liquid chromatography–tandem mass spectrometry (UHPLC-MS/MS); however, degradation products and impurities were not evaluated. Additionally, the results may not be directly extrapolated to other drug concentrations, alternative diluents such as D5W, or different container types, including polyolefin or polypropylene infusion bags and silicone elastomeric pumps. Furthermore, although stability at 25, 32, and 37 °C was evaluated for up to 48 h, longer exposure at ambient temperatures was not investigated. Future studies could provide valuable information on drug stability following prolonged breaks in the cold chain or other unanticipated delays before administration.

Another potential limitation of this study is the definition of chemical stability. Stability was defined as the retention of at least 90% of the initial drug concentration, a criterion commonly used in pharmaceutical stability studies. However, some regulatory guidelines, such as the Yellow Cover Document (YCD) of the United Kingdom’s National Health Service (NHS), recommend a more stringent acceptance range of 95–105% [18]. According to this criterion, ATM stored at 37 °C for 48 h would not be considered chemically stable, despite remaining above the 90% threshold adopted in this study. Therefore, the interpretation of these findings should take into account the stability acceptance criterion applied.

To our knowledge, this is the first study to evaluate the physicochemical stability of ATM/AVI under clinically relevant conditions. Given the increasing role of this combination in the treatment of infections caused by MDR Gram-negative pathogens, these findings provide valuable data for its use in OPAT programs.

## 4. Materials and Methods

### 4.1. Materials

Avibactam sodium (AVI), aztreonam (ATM), and ampicillin (AMP) standards were purchased from Eurodiagnostico S.L. (Madrid, Spain). AVI and ATM were used for UHPLC-MS/MS calibration and quantification, whereas AMP was used as the internal standard.

Pharmaceutical doses were prepared from the commercially available intravenous formulation Emblaveo (ATM/AVI 1.5/0.5 g powder for the concentrate for solution for infusion) purchased from Pfizer Laboratories (Madrid, Spain). Each vial contained 1.5 g of aztreonam and sodium avibactam equivalent to 0.5 g of avibactam.

Sterile water for injection used for the reconstitution of the drug vials was purchased from Serra Pamies Laboratories (Tarragona, Spain). NS bags were purchased from Grifols (Barcelona, Spain).

For the preparation of the solution tests, drugs were stored in polyisoprene elastomeric pumps that were supplied by Baxter Healthcare S.A. (Zurich, Switzerland).

Liquid chromatography–mass spectrometry (LC-MS)-grade methanol was obtained from Fisher Scientific S.L. (Madrid, Spain). Ammonium acetate 97% ACS Reagent was obtained from Merck Life Sciences S.L.U. (Madrid, Spain). Purified water was obtained using a Milli-Q Advantage A10 ultrapure water system (Merck Life Science S.L.U., Madrid, Spain).

### 4.2. Preparation of ATM/AVI Solutions

The ATM/AVI (1.5 g/0.5 g) vial was reconstituted with 10 mL of water for injection. This solution was diluted with NS to obtain a final concentration of 25 mg/mL aztreonam and 8.33 mg/mL avibactam. Three elastomeric infusion devices (Infusor LV 10 mL/h, 240 mL; Baxter Healthcare S.A) were prepared for each temperature condition.

### 4.3. Storage Conditions and Sampling

Elastomeric devices were stored at four different temperatures: 4 ± 2 °C, 25 ± 2 °C, 32 ± 2 °C, and 37 ± 2 °C. For devices maintained under refrigerated conditions (4 ± 2 °C), samples were collected for analysis over a 14-day period (0 h, 24 h, 48 h, 72 h, day 7, and day 14). For devices stored at 25 ± 2 °C, 32 ± 2 °C, and 37 ± 2 °C, samples were taken over 48 h (0 h, 24 h, and 48 h). Storage temperatures under refrigerated and room-temperature conditions were monitored using a data logger. Samples stored at 32 °C and 37 °C were maintained in a temperature-controlled incubator to ensure stable storage conditions throughout the study.

No additional protection from light was used during storage, as the elastomeric infusion devices provide protection against UVB, UVC, and most UVA radiation.

At each time point, duplicate samples were collected from each preparation and frozen at −80 °C until the analysis. Frozen storage of ATM and AVI samples prior to analysis has been previously described and supported in the literature [9,19]. Prior to chemical analysis, samples were diluted in Milli-Q water, vortexed, and aliquoted into autosampler vials. The internal standard (AMP) was added, and samples were injected into the UHPLC-MS/MS system.

### 4.4. UHPLC-MS/MS Quantification

Antibiotic concentrations were measured using an ultra-high-performance liquid chromatography–tandem mass spectrometry (UHPLC-MS/MS) method. Samples were analyzed using an UHPLC Elute+ coupled to a QTOF Compact mass spectrometer (Bruker Española S.A., Madrid, Spain) in negative ionization mode using electrospray. Nitrogen was used as the collision gas. AMP was used as the internal standard for AVI and ATM. For chromatographic separation, a BRHSC18022100 Bruker Intensity Solo C18 column, 2.0 × 100 mm, 2.0 µm (Bruker Española S.A., Madrid, Spain), was used. The UHPLC coupled to quadrupole time-of-flight mass spectrometry (UHPLC-qTOF-MS) method provides high-resolution, accurate-mass detection, enabling discrimination of the parent compounds from potential degradation products with similar mass-to-charge (*m*/*z*) ratios. Full-scan mass spectrometry (MS) and broadband collision-induced dissociation (bbCID) acquisition enable retrospective analysis of all detected ions and their corresponding fragment ions, further supporting the stability-indicating capability of the method [19,20]. Chromatographic and mass spectrometry conditions are described in Table S1 (Supplementary Material). Representative chromatograms and mass spectra confirming antibiotic stability are provided in the Supplementary Material (Figures S1–S5).

### 4.5. Chemical Stability

Drug stability was calculated as the percentage (P) of the initial drug remaining in the device at each analyzed time point (C_t_), in relation to the initial concentration (C_0_) (P = C_t_/C_0_ × 100). Chemical stability was defined as the recovery of more than 90% of the initial antibiotic concentration. Data were expressed as mean ± standard deviation (SD).

### 4.6. Physical Stability

Color change, clarity, and precipitation were assessed via visual inspection at each sampling time point across all storage conditions. Evaluation was based on the criteria established in the Emblaveo^®^ Summary of Product Characteristics: clear, colorless to yellow solution, and no visible particles [7].

The pH was determined at each analysis point using a HANNA^®^ HI 2221 pH meter. A variation of more than one pH unit from the initial measurement was considered an indicator of physical instability. pH values were expressed as mean ± standard deviation (SD).

## 5. Conclusions

ATM/AVI remained physicochemically stable in polyisoprene elastomeric devices for up to 14 days under refrigerated conditions (4 °C) and for 48 h at 25 °C, 32 °C, and 37 °C. These findings support the potential use of ATM/AVI for continuous infusion in OPAT programs under the specific conditions evaluated, pending consideration of microbiological stability, device type, drug concentration, and local aseptic preparation standards.

## Figures and Tables

**Table 1:** Chemical stability at 4 °C, 25 °C, 32 °C, and 37 °C.

		Concentration Remaining (Mean ± SD)	
Temperature (°C)	Time	ATM	AVI
4	24 h	101.66 ± 5.28	95.78 ± 4.95
	48 h	101.14 ± 3.23	101.76 ± 6.33
	72 h	102.29 ± 6.39	100.20 ± 7.50
	7 days	101.15 ± 8.86	101.08 ± 6.67
	14 days	97.17 ± 6.50	98.79 ± 5.65
25	24 h	101.43 ± 2.83	104.72 ± 4.50
	48 h	100.26 ± 3.08	105.16 ± 6.47
32	24 h	98.26 ± 3.91	98.02 ± 2.58
	48 h	97.13 ± 3.16	98.73 ± 5.93
37	24 h	95.23 ± 2.78	99.69 ± 5.31
	48 h	93.85 ± 1.71	100.06 ± 6.97

ATM: aztreonam; AVI: avibactam; SD: standard deviation. For each temperature condition, three independent elastomeric infusion devices were prepared. At each time point, duplicate samples were collected from each device.

**Table 2:** Physical stability at 4 °C, 25 °C, 32 °C, and 37 °C.

Temperature (°C)	Time	Color	Clarity	Precipitation	pH (Mean ± SD)
4	0 h	Colorless	Yes	No	4.99 ± 0.01
	24 h	Colorless	Yes	No	5.03 ± 0.02
	48 h	Colorless	Yes	No	5.03 ± 0.03
	72 h	Colorless	Yes	No	5.04 ± 0.04
	Day 7	Colorless	Yes	No	4.93 ± 0.05
	Day 14	Colorless	Yes	No	4.93 ± 0.02
25	0 h	Colorless	Yes	No	5.00 ± 0.00
	24 h	Colorless	Yes	No	5.03 ± 0.01
	48 h	Colorless	Yes	No	5.06 ± 0.01
32	0 h	Colorless	Yes	No	5.01 ± 0.03
	24 h	Colorless	Yes	No	4.97 ± 0.01
	48 h	Colorless	Yes	No	5.04 ± 0.09
37	0 h	Colorless	Yes	No	4.99 ± 0.01
	24 h	Colorless	Yes	No	5.06 ± 0.02
	48 h	Colorless	Yes	No	5.08 ± 0.01

SD: standard deviation.

## Data Availability

The raw data supporting the conclusions of this article will be made available by the authors on request.

## Supplementary Materials

The following supporting information can be downloaded at: https://www.mdpi.com/article/10.3390/antibiotics15070708/s1, Table S1. Chromatographic and mass spectrometry conditions; Figure S1. Representative base peak chromatograms (BPC) of Avibactam, Aztreonam, and Ampicillin (internal standard) at (a) time zero and at the end of the monitoring period: (b) 4 °C for 14 days, (c) 25 °C for 48 h, (d) 32 °C for 48 h, and (e) 37 °C for 48 h. Raw unprocessed data acquired using UHPLC-MS (top) and UHPLC-bbCID-MS (bottom). Figure S2. Comparison of the MS spectra for the peaks observed in the chromatograms shown in the upper panels (a and e) of Figure S1 at (a) time zero and (b) after 48 h at 37 °C. Figure S3. Comparison of the bbCID-MS spectra for the peaks observed in the chromatograms shown in the lower panels (a and e) of Figure S1 at (a) time zero and (b) after 48 h at 37 °C. Figure S4. Representative base peak chromatograms (BPC) of Avibactam, Aztreonam, and Ampicillin (internal standard) at different time points during storage at 25 °C. Raw unprocessed data acquired using UHPLC-MS (top) and UHPLC-bbCID-MS (bottom). Figure S5. Representative base peak chromatograms (BPC) of avibactam, aztreonam, and ampicillin (internal standard) at different time points during storage at 4 °C. Raw unprocessed data acquired using UHPLC-MS (top) and UHPLC-bbCID-MS (bottom).

## Abbreviations

The following abbreviations are used in this manuscript:

OPAT	Outpatient Parenteral Antimicrobial Therapy
PK/PD	Pharmacokinetic/pharmacodynamic
MIC	Minimum Inhibitory Concentration
ATM	Aztreonam
AVI	Avibactam
ATM/AVI	Aztreonam/avibactam
MDR	Multidrug-resistant
D5W	5% Dextrose
AMP	Ampicillin
NS	Normal saline (0.9% sodium chloride solution)
CI	Confidence Interval
UHPLC-MS/MS	Ultra-High-Performance Liquid Chromatography–Tandem Mass Spectrometry
SD	Standard Deviation
