# Supplementary material for: Physicochemical Stability of Aztreonam/Avibactam in Elastomeric Devices for Outpatient Parenteral Antimicrobial Therapy
Source: Antibiotics (Basel). 2026 Jul 21;15(7):708. doi: 10.3390/antibiotics15070708 (PMC13405842; doi:10.3390/antibiotics15070708)

SUPPLEMENTARY MATERIAL

Table S1. Chromatographic and mass spectrometry conditions

Cromatographic conditions:<br><br>The mobile phase consisted of Water:Methanol (99:1) with 5 mM ammonium acetate (phase A) and 5 mM ammonium acetate in methanol (phase B). Gradient: 0-0.2min: 4%B. 0.2-2min: gradual increase until 20%B. 2-4.5min: gradual increase until 70%B. 4.5-5.5min: 70%B. 5.50-5.51min: direct increase to 100%B. 5.51-6.51min 100%B. 6.51-7.00min: decrease to 4%B. 7.00-9.00min: 4%B. The column and auto-sampler tray temperatures were set at 25ºC and 4ºC, respectively. Flow rate was set at 0.3 mL/min.
Mass spectrometry conditions:<br><br>Combination of MS and bbCID MS (bbCID (MS-MS/MS) isCID Energy MS: 0.0-5.0eV and Collision Energy MS: 6.0-30.0eV) methods were used for quantitation and qualification, respectively. Monitored ions: MS (bbCID): Avibactam: $C_7H_{10}N_3O_6S^{-1}$ (SO_4^{-1}), Aztreonam: $C_{13}H_{16}N_5O_8S_2^{-1}$ ($C_2H_4NO_3S^{-1}$) i Ampicillin: $C_{16}H_{18}N_3O_4S^{-1}$ ($C_{10}H_{11}N_2OS^{-1}$).

Figure S1. Representative base peak chromatograms (BPC) of Avibactam, Aztreonam, and Ampicillin (internal standard) at (a) time zero and at the end of the monitoring period: (b) 4°C for 14 days, (c) 25°C for 48 h, (d) 32°C for 48 h, and (e) 37°C for 48 h. Raw unprocessed data acquired using UHPLC-MS (top) and UHPLC-bbCID-MS (bottom).

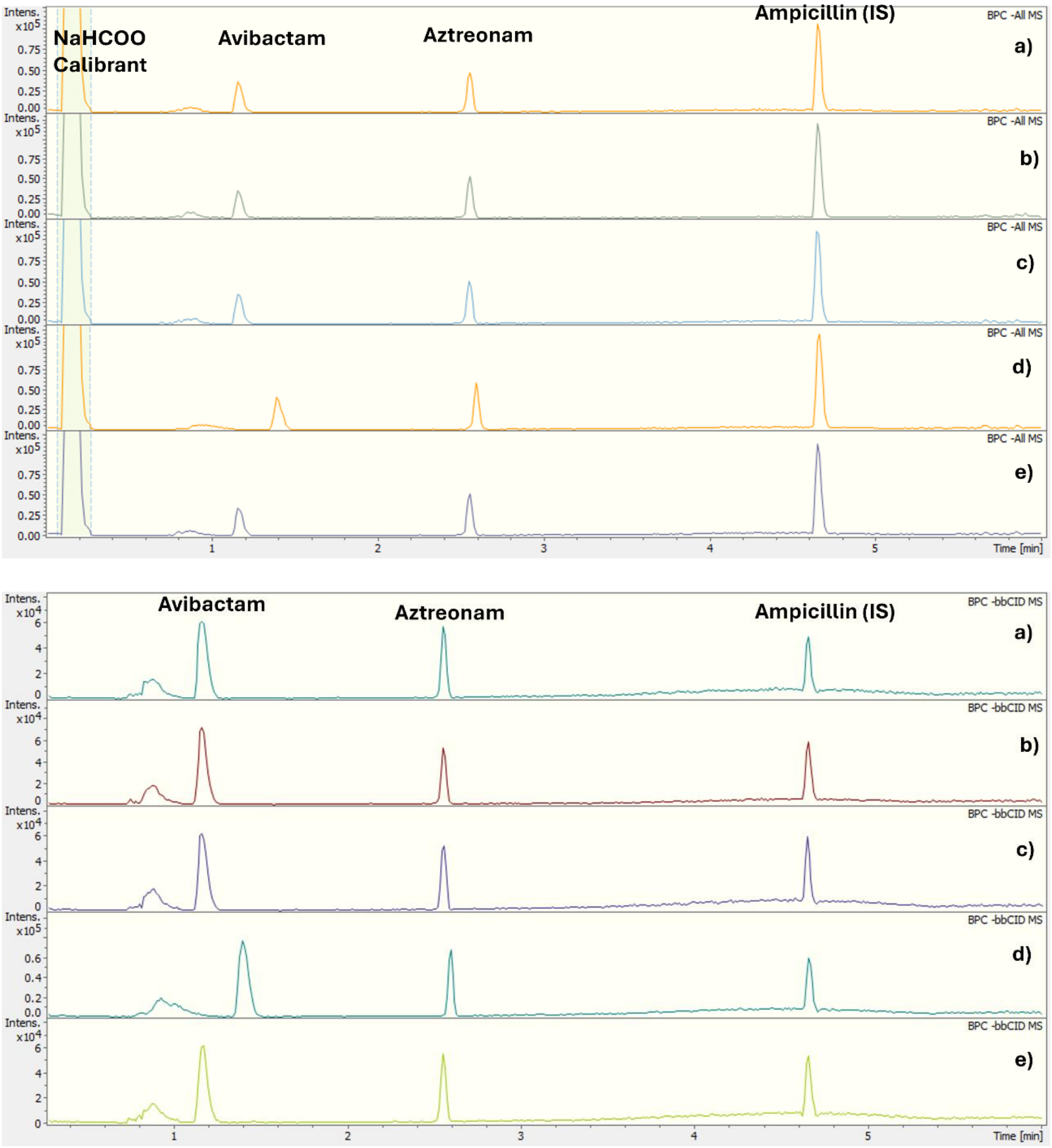

Figure S2. Comparison of the MS spectra for the peaks observed in the chromatograms shown in the upper panels (a and e) of Figure S1 at (a) time zero and (b) after 48 h at 37°C.

a)

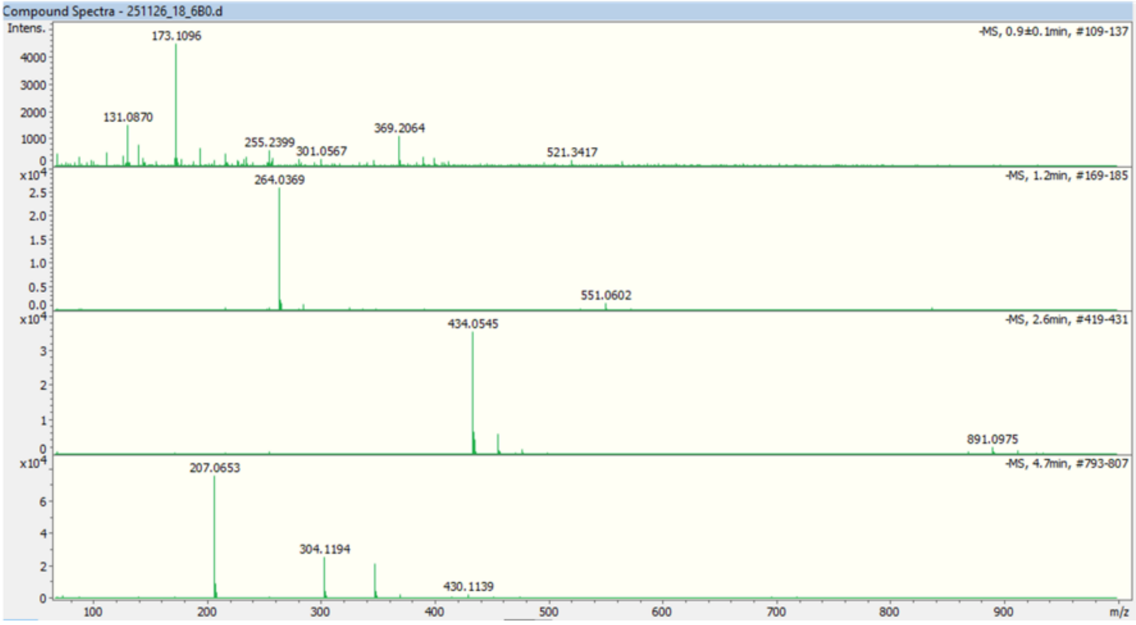

b)

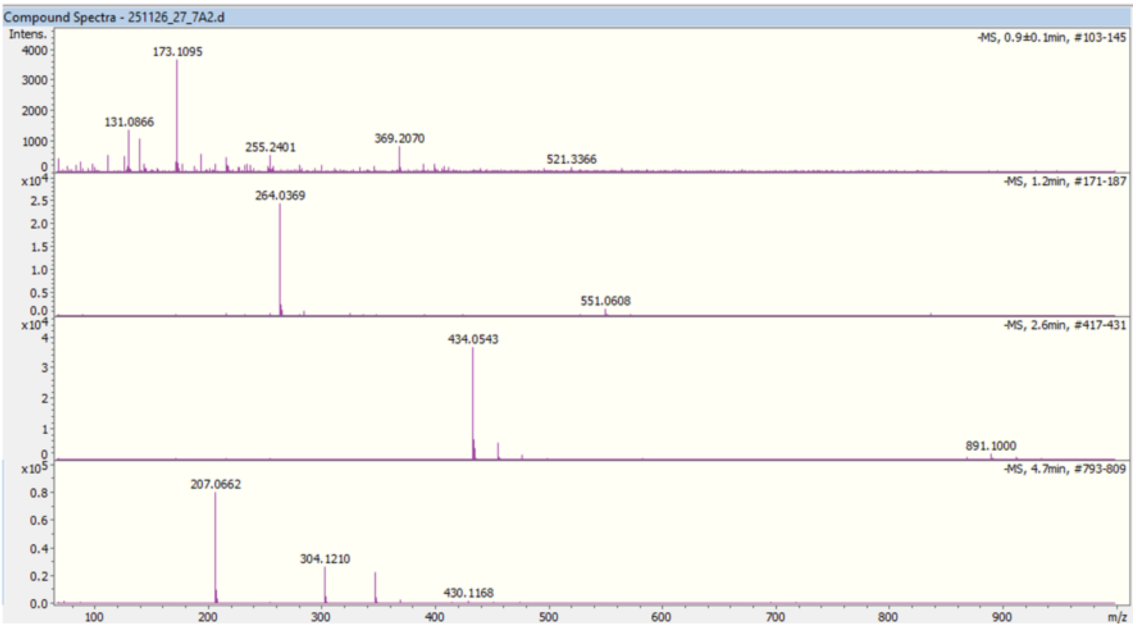

Figure S3. Comparison of the bbCID-MS spectra for the peaks observed in the chromatograms shown in the lower panels (a and e) of Figure S1 at (a) time zero and (b) after 48 h at 37°C.

a)

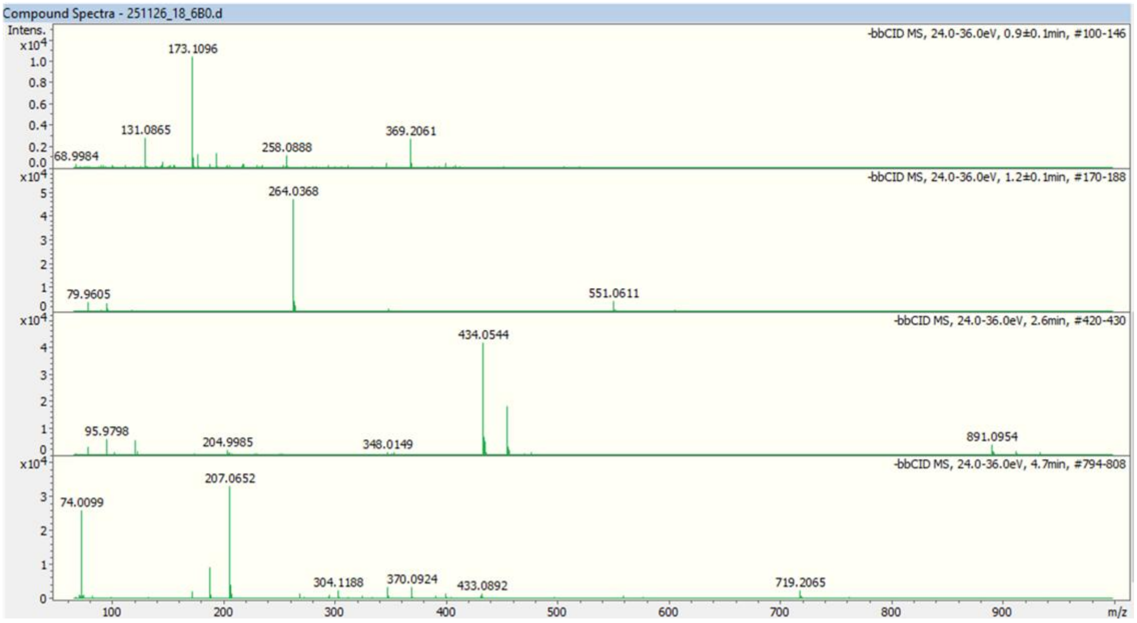

b)

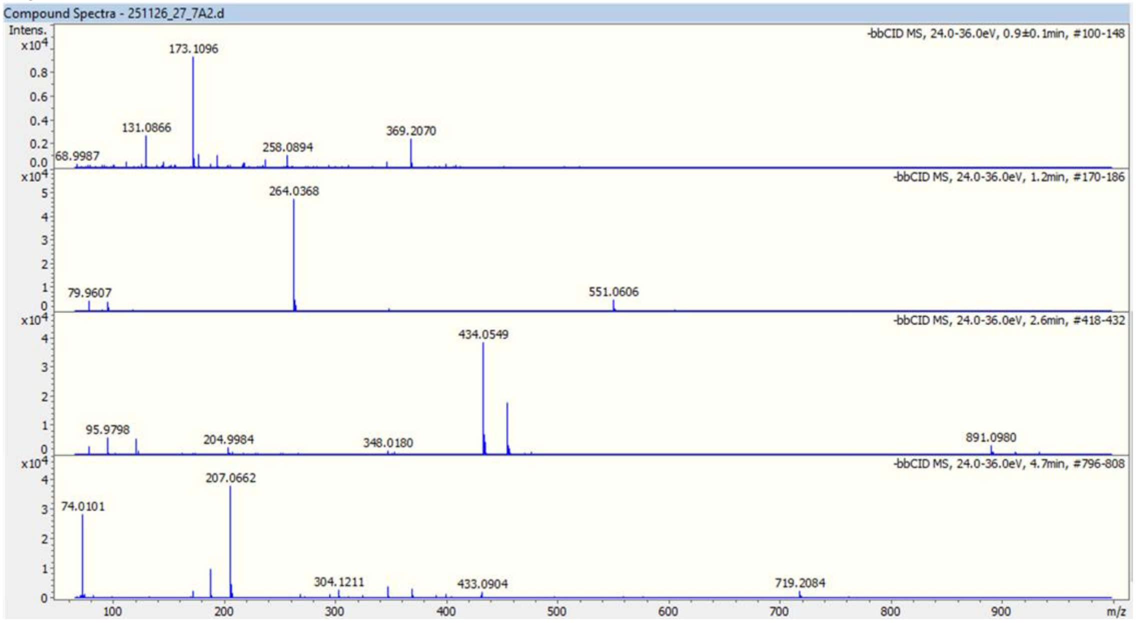

Figure S4. Representative base peak chromatograms (BPC) of Avibactam, Aztreonam, and Ampicillin (internal standard) at different time points during storage at 25°C. Raw unprocessed data acquired using UHPLC-MS (top) and UHPLC-bbCID-MS (bottom).

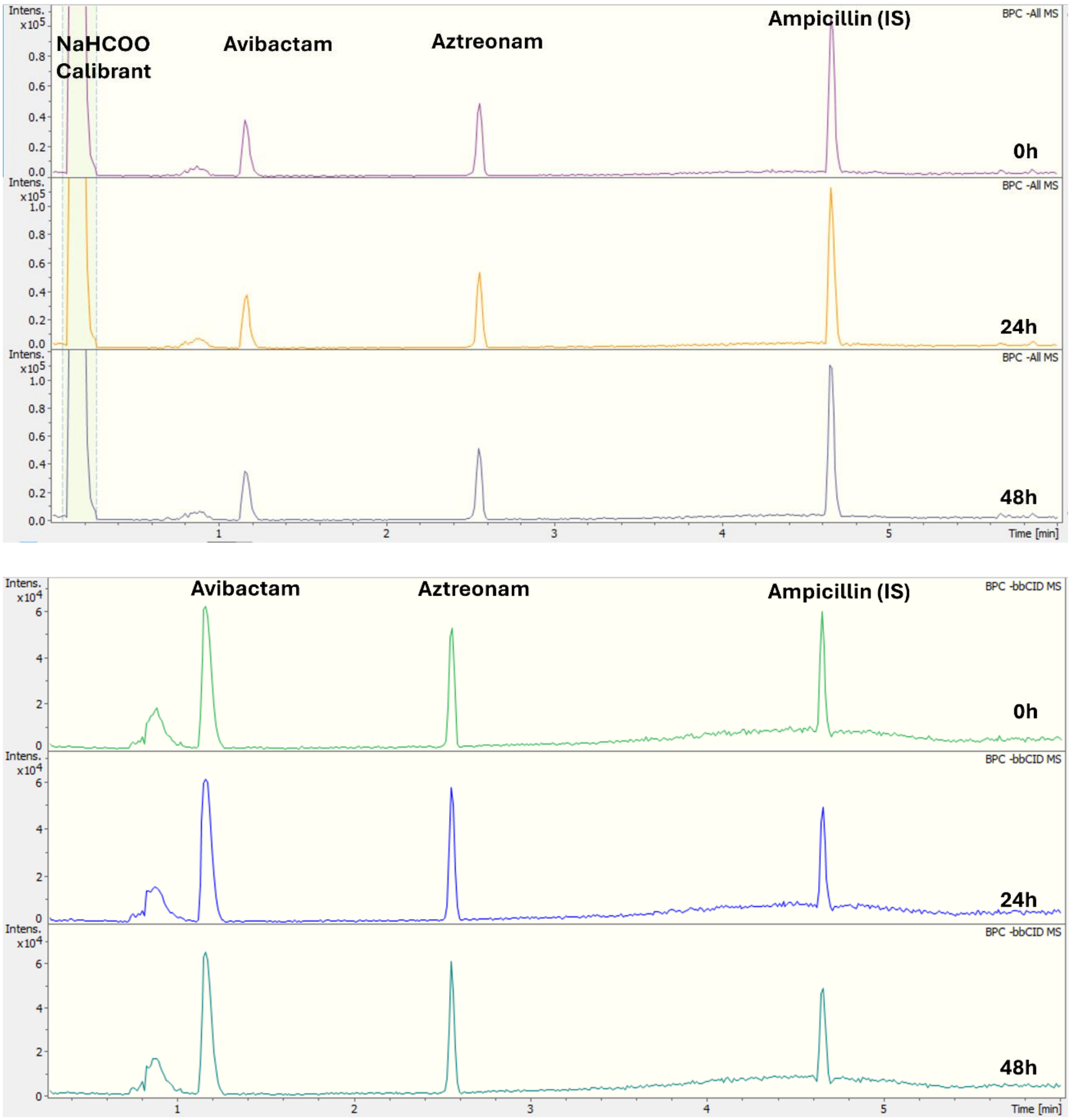

Figure S5. Representative base peak chromatograms (BPC) of avibactam, aztreonam, and ampicillin (internal standard) at different time points during storage at 4°C. Raw unprocessed data acquired using UHPLC-MS (top) and UHPLC-bbCID-MS (bottom).

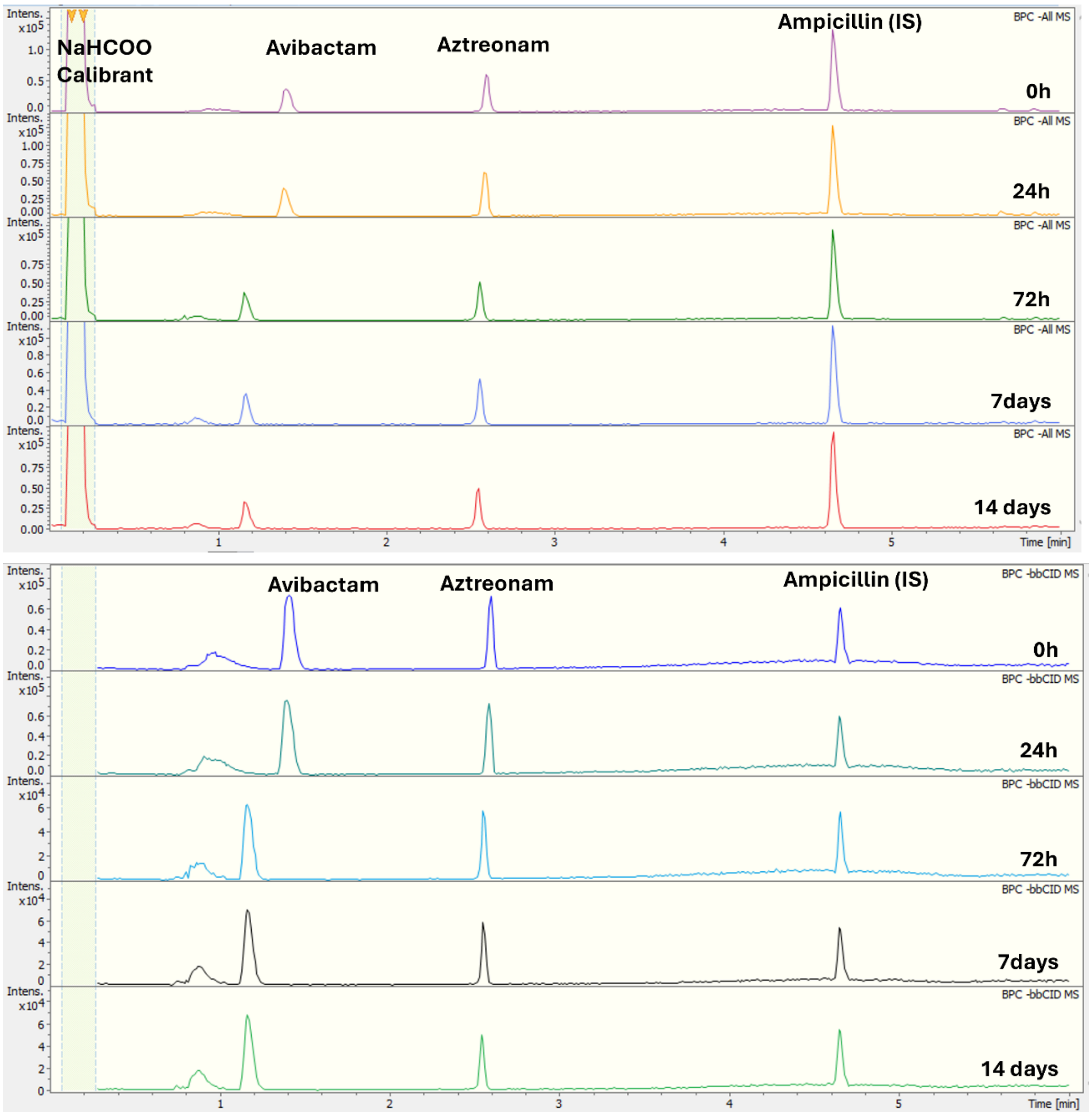

Supplement: Supplementary file 1 [file antibiotics-15-00708-s001.zip › antibiotics-4412395-supplementary.pdf]
